# Noble Metal Complexes and Non-Canonical Nucleic Acids: From G-Quadruplex Recognition to Emerging Functional Architectures

**DOI:** 10.3390/biom16060835

**Published:** 2026-06-05

**Authors:** Damiano Cirri, Alessandro Pratesi

**Affiliations:** Department of Chemistry and Industrial Chemistry, University of Pisa, Via Giuseppe Moruzzi 13, 56124 Pisa, Italy; damiano.cirri@unipi.it

**Keywords:** G-quadruplex, i-motif, Z-DNA, Z-RNA, R-loops, junctional DNA, RNA structures, platinum, gold, palladium, silver, metallodrugs, proteomics, metabolomics, metallomics

## Abstract

Non-canonical nucleic acid structures such as G-quadruplexes (G4s), i-motifs, triplexes, junctions, and structured RNA domains offer coordination environments that differ fundamentally from those of canonical duplex DNA. This review is deliberately G4-centred, because DNA G4s currently provide the most mature mechanistic and biological evidence for noble-metal recognition, while i-motifs, quadruplex–duplex hybrids, junctional structures, R-loops, Z-DNA/Z-RNA, and structured RNA remain emerging or less extensively validated target classes. The discussion addresses how platinum, gold, palladium, and silver complexes recognize these architectures through combinations of coordination chemistry, pi-stacking, electrostatics, scaffold-dependent shape complementarity, and metal-mediated base pairing. A further distinction is made between direct structural recognition, cellular target engagement, and downstream phenotypic responses, emphasizing where causality has been experimentally demonstrated and where it remains inferential. Particular emphasis is placed on G-quadruplexes in telomeric, promoter, and mitochondrial contexts, while i-motifs, junctional DNA, hybrid DNA/RNA structures, and structured RNA are treated as expanding but less mature areas of investigation. The review also critically addresses selectivity, resistance, delivery, and translational challenges, highlighting how the concept of functional architectures can help unify structural chemistry with pathway-level biology in the design of next-generation metallodrugs.

## 1. Introduction

Nucleic acids are no longer regarded as passive carriers of genetic information, but rather as structurally versatile macromolecules capable of adopting a wide range of non-canonical conformations with distinct chemical and biological properties. Beyond the classical B-form DNA and A-form RNA, structures such as G-quadruplexes (G4s), i-motifs, triplexes, mismatches, bulges, junctions, and complex RNA folds play regulatory roles in replication, transcription, translation, and genome stability [1]. These alternative conformations expose unconventional binding pockets and coordination sites that are poorly accessible in canonical duplexes, thereby offering unique opportunities for selective chemical functionalization and metal coordination [2,3,4].

In this context, the metalation of non-canonical DNA and RNA structures has emerged as a rapidly expanding field at the interface of coordination chemistry, structural biology, medicinal inorganic chemistry, and nanoscience. Metalation may involve the direct coordination of metal ions to nucleobase donor atoms, metal-mediated base pairing, or non-covalent recognition driven by π-stacking, electrostatics, and shape complementarity [3,5]. In particular, transition metal complexes provide unique opportunities to couple structural recognition with tunable physicochemical properties such as redox activity, photophysics, and ligand exchange kinetics.

What makes this area particularly compelling for medicinal chemistry is that topology and local electronics are intimately intertwined: the same complex can behave differently depending on whether it encounters a duplex major groove, a terminal G-tetrad, a cytosine-rich i-motif pocket, or an RNA internal loop. This context-dependence is especially evident for noble metal complexes. Soft metal ions such as Au(I) and Ag(I) tend to form reversible, often non-covalent interactions with nucleobases and secondary structures, whereas square-planar d^8^ metal centers such as Pt(II) and Pd(II) can engage in more persistent interactions, including stacking, groove binding or, in some cases, covalent coordination [6,7].

**Figure 1:**
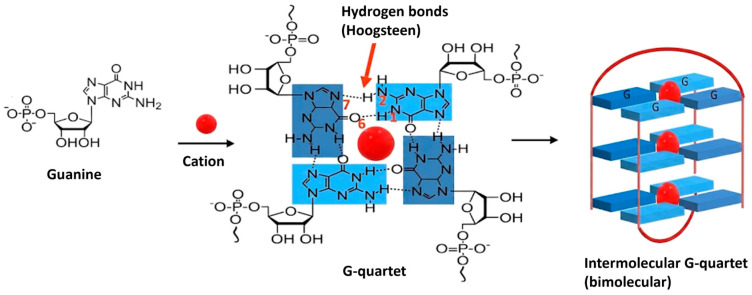
G-quartet and a possible G-quadruplex structure. Reproduced from Reference [4] under a Creative Commons CC-BY license.

Ligand design plays a decisive role in dictating these behaviors. π-Extended aromatic scaffolds promote end-stacking on G-tetrads, cationic substituents enhance interactions with the phosphate backbone and loop regions, and multimetallic or multivalent architectures can target higher-order nucleic acid assemblies. For example, dimeric and multinuclear metal complexes have been shown to selectively recognize multimeric G4 structures or bridge adjacent quadruplex units, leading to enhanced selectivity compared to monomeric analogues [8]. Likewise, organometallic Au(I) *N*-heterocyclic carbene complexes can stabilize G4 structures predominantly through non-covalent interactions, highlighting an alternative paradigm to classical DNA cross-linking mechanisms (Figure 1) [9].

**Figure 2:**
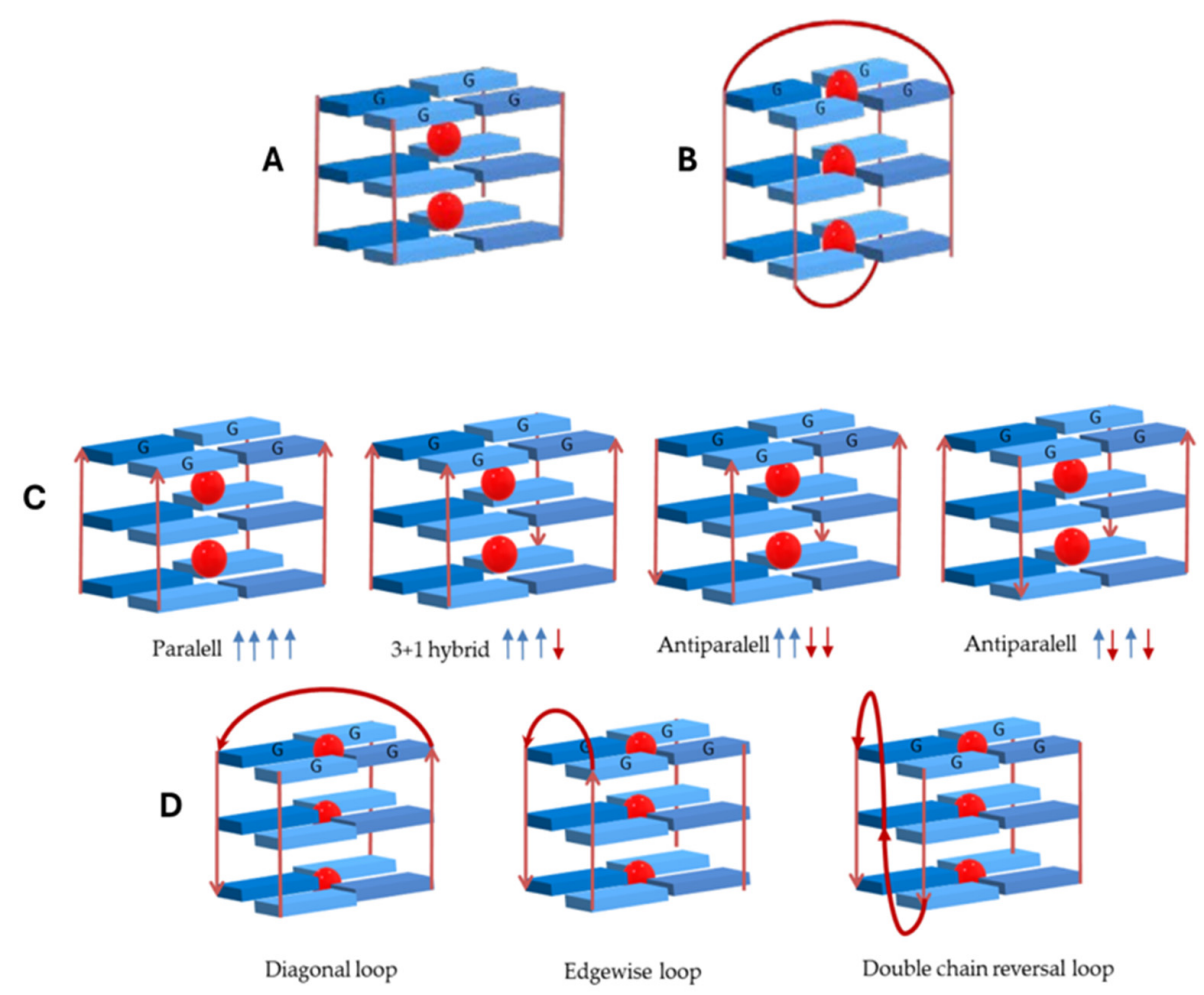
Structural features and conformational variability of G-quadruplexes. (**A**) Positioning of stabilizing cations between stacked G-quartets and (**B**) within the plane of the tetrads in a three-quartet G-quadruplex (sugar–phosphate backbones omitted for clarity). (**C**) Representative G-quadruplex conformations arising from different strand orientations, and (**D**) common loop geometries contributing to topological diversity. Reproduced from Reference [4] under a Creative Commons CC-BY license.

Among the non-canonical DNA targets, G4 structures (Figure 2) have received the greatest attention due to their presence in telomeres and oncogene promoter regions and their direct involvement in cancer-related processes. Stabilization of G4 structures can interfere with telomerase activity or transcriptional regulation, providing a mechanistic basis for anticancer activity [10,11]. This is exemplified by a wide range of Pt(II), Au(I), and Pd(II) complexes that selectively bind and stabilize G4 DNA through stacking or groove interactions [6,7]. However, the field is increasingly moving beyond a purely G4-centered perspective. I-motifs are now recognized as dynamic regulatory elements rather than in vitro curiosities, and RNA offers an even richer structural landscape due to its higher conformational plasticity and functional diversity [1,3].

A major conceptual shift has also occurred at the biological level. Early studies on metal–nucleic acid interactions primarily focused on binding affinity and structural characterization. More recent work instead emphasizes how specific recognition events translate into functional outcomes. For instance, stabilization of promoter G4s can modulate gene expression, while targeting mitochondrial G4 structures can impair mitochondrial DNA replication, transcription, and protein synthesis, ultimately leading to mitochondrial dysfunction and altered cellular energetics [12].

This broader perspective is further reinforced by the integration of omics-based approaches, including proteomics and metabolomics, which reveal that the consequences of metal–nucleic acid interactions extend well beyond the primary target. Changes in nucleic acid structure can propagate into alterations in redox homeostasis, cytoskeletal organization, mitochondrial function, and stress-response pathways, as demonstrated for gold-based G4 stabilizers with multimodal mechanisms of action [9].

**Scheme 1:**
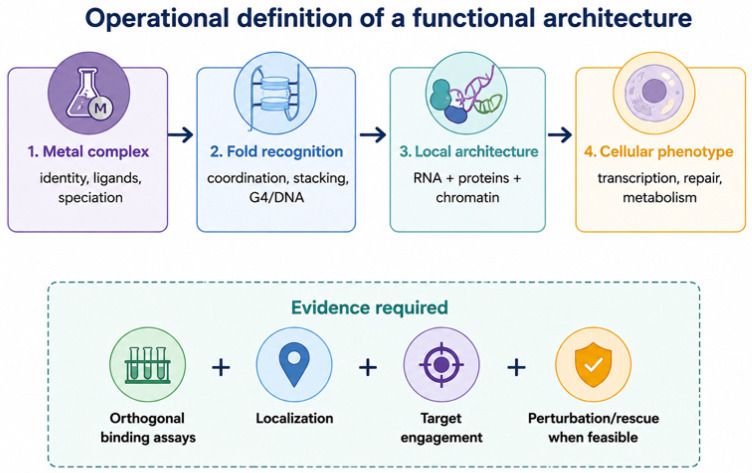
Operational model linking noble-metal speciation and ligand design to fold recognition, local nucleic-acid-centered architecture, and downstream cellular phenotype.

Accordingly, the most appropriate framework for this field is no longer limited to ‘binding to a nucleic acid structure’ but rather involves the formation of functional architectures in which metal complexes, nucleic acid folds, proteins, and metabolic pathways are mechanistically interconnected (Scheme 1). In this review, noble metal coordination to non-canonical DNA and RNA structures is discussed from this integrated perspective. However, the strength of the available evidence is not uniform across target classes. At present, the most robust mechanistic and biological dataset concerns DNA G-quadruplexes, whereas i-motifs, quadruplex–duplex hybrids, junctional architectures, and structured RNA remain promising but less extensively validated targets.

In this review, the term functional architecture is used to describe the broader molecular context in which folded DNA or RNA structures interact with metal complexes. This context includes associated proteins, local cellular compartmentalization, and the chemical form of the metal species, all of which can influence the final biological response. Direct binding to an isolated oligonucleotide is therefore necessary but not sufficient to claim perturbation of a functional architecture. Stronger evidence requires a graded set of observations: selective binding to the relevant fold; persistence of the active species in biological media; cellular or subcellular access to the appropriate compartment; perturbation of local nucleic-acid–protein interactions or transcriptional/replicative readouts; and, where feasible, competition, genetic, chemical, or rescue experiments linking the phenotype to the targeted structure rather than to generic metal-induced stress.

Within this framework, the structural landscape of major non-canonical targets and the principles governing their recognition by Pt, Au, Pd, and Ag complexes are first examined. Dominant binding modes and determinants of selectivity are then analyzed, before addressing how local recognition events may translate into transcriptional regulation, replication stress, mitochondrial dysfunction, and broader cellular responses. Finally, current limitations and future directions for the rational design of next-generation metallodrugs targeting structured nucleic acids are highlighted.

### Scope, Selection Criteria and Evidence Grading

In this frame, this article should therefore be read as a selective, perspective-driven review rather than as an exhaustive catalogue of every metal–nucleic acid interaction. The literature was selected to prioritize studies in which Pt, Au, Pd or Ag complexes were examined against non-canonical DNA or RNA structures using at least one structure-sensitive method, preferably in combination with selectivity assays, cellular localization, target-engagement experiments, or systems-level readouts (Table 1).

**Scheme 2:**
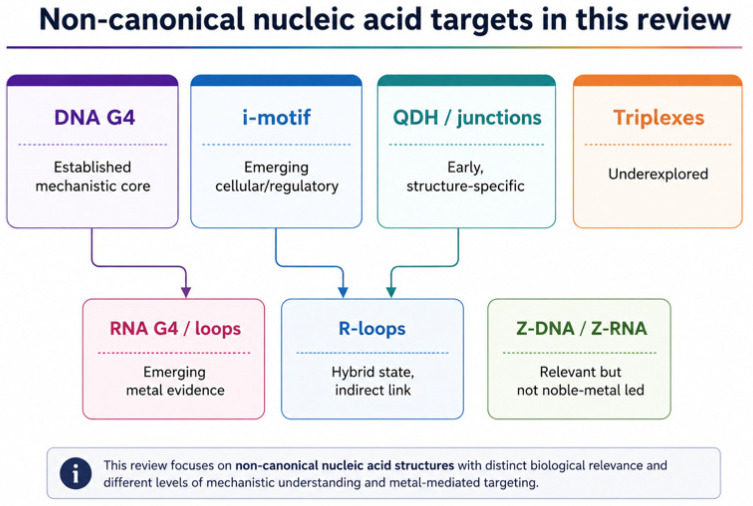
Schematic overview of the target classes considered in this review and their approximate evidence of maturity for noble-metal recognition. This latter decreases from direct structural and cellular target engagement to indirect or mostly contextual evidence.

The emphasis on Pt, Au, Pd, and Ag reflects both the medicinal relevance of platinum-family coordination chemistry and the distinctive soft-metal, organometallic, redox, photophysical, and metal-mediated pairing behavior of gold and silver compounds. To address the uneven maturity of the field, the discussion explicitly distinguishes established mechanisms, mostly DNA G4 systems supported by orthogonal biophysical and cellular evidence, from emerging or inferred cases involving i-motifs, RNA structures, R-loops, Z-DNA/Z-RNA, triplexes, and junctional architectures (Scheme 2).

The organization of this paper has been adjusted accordingly. Section 2 and Section 3 focus on structural and physicochemical recognition; Section 4 discusses biological consequences only when the experimental chain from binding to phenotype can be assessed; Section 5 treats omics, imaging, and interactome data as evidence layers rather than as automatic proof of target engagement; and Section 6 is restricted to design principles that are directly relevant to metal complexes or metal-enabled probes.

## 2. Molecular Recognition of Non-Canonical DNA and RNA Structures

The concept of molecular recognition is particularly useful in noble metal–nucleic acid chemistry because non-canonical structures are not generic DNA- or RNA-like objects, but topological entities with distinct stereoelectronic signatures. Recognition depends on base composition, folding topology, loop geometry, hydration shell, electrostatics, cation occupancy, and conformational dynamics (Table 2). As a result, a metal complex that binds only modestly to duplex DNA may become highly competent toward a G4 if its ligand framework complements the exposed aromatic surface of a terminal tetrad or accesses donor atoms that are not available in canonical duplexes [5,13,14].

A second defining feature of these targets is their intrinsic structural dynamics. Recognition should therefore be viewed not as a single lock-and-key event, but as selection within an ensemble of states, where a metal complex may function as a binder, topological selector, folding chaperone, or misfolding inducer [1]. In practice, however, these recognition paradigms are supported to very different extents depending on the target class, with DNA G-quadruplexes providing by far the clearest and most extensively characterized case.

### 2.1. G-Quadruplex DNA as a Privileged Target

G4 DNA remains the most intensively investigated non-canonical target for noble metal complexes because telomeric and promoter G4s lie at the intersection of structural accessibility and biological relevance. Telomeric G4s are associated with chromosome-end protection and telomerase regulation, whereas promoter G4s modulate oncogene expression, including MYC, KIT, and BCL2 [11,15,20].

Among platinum-based systems, the progression from simple square-planar scaffolds to more elaborate architectures illustrates how ligand design governs recognition. Pt(II)-phenanthroline derivatives show that increasing methyl substitution enhances G4 affinity relative to duplex DNA, linking aromatic surface expansion to improved recognition [21]. Cyclometallated Pt(II) complexes stabilize both c-MYC and telomeric G4s while retaining luminescence, whereas water-soluble Pt(II) luminophores demonstrate that substituent identity and solubilizing patterns modulate binding behavior beyond simple planarity [6,10].

Topological selectivity becomes more explicit in multinuclear systems. Fan-shaped trinuclear Pt(II) complexes such as {[Pt(dien)]_3_(tib)}^6+^ and {[Pt(dpa)]_3_(tib)}^6+^ display a strong preference for telomeric G4s over promoter G4s and duplex DNA, induce antiparallel folding, and inhibit telomerase activity [7]. Similarly, dimeric metal–salphen systems preferentially recognize multimeric telomeric G4 assemblies, highlighting the importance of higher-order structural organization [22].

Promoter G4s provide a stringent test of fine selectivity. In the c-MYC system, organoplatinum N^N^C complexes derived from the 2-([2,2′-bipyridin]-6-yl)phenyl scaffold consistently prefer c-MYC over telomeric and duplex DNA, but their performance depends strongly on side-chain identity. Morpholine-substituted derivatives achieve the highest stabilization, while imide and heteroaryl variants demonstrate how loop and cap interactions reshape both binding affinity and optical response [2,23]. These observations emphasize that end-stacking and peripheral interactions must be considered together.

Structural studies provide further insight into recognition mechanisms. The Pt-phen complex bound to the MYC G4 forms a sequential 1:1 and then 2:1 adduct, occupying first the 3′ and then the 5′ end, with extensive π-stacking and rearrangement of flanking residues. This illustrates that G4 binding can be directional, adaptive, and stoichiometry-dependent [24].

Gold complexes complement this picture through predominantly non-covalent recognition. The bis-NHC Au(I) complex AuTMX_2_ is a benchmark system combining G4 selectivity with a biologically relevant scaffold [9,25]. Dinuclear Au(I) bis-NHC macrocycles show how linker length and flexibility modulate productive interaction with terminal tetrads [26], while Au(III) porphyrins extend recognition to larger aromatic platforms where charge distribution and substitution pattern control selectivity [20,27].

Telomeric systems further reveal the adaptive nature of metal binding. The Au(III)-TPymT-α complex bound to Tel24 occupies multiple closely related positions within the same binding region, rather than adopting a single rigid pose, underscoring the importance of conformational heterogeneity [28].

Recognition is also expanding beyond nuclear DNA. The Pt-ttpy complex accumulates in mitochondria, perturbs mtDNA, suppresses mitochondrial transcription and translation, and affects nuclear genes enriched in promoter G4 motifs, linking quadruplex recognition to mito-nuclear communication [12]. In parallel, rDNA and rRNA G4s are emerging as nucleolar targets, with predominantly parallel topologies supported by combined spectroscopic and computational analyses [29].

Overall, these studies show that affinity or ΔTm values are useful entry points but not sufficient descriptors of recognition quality. In the revised evidence framework, a G4-directed noble-metal complex is considered strongly supported only when structure-sensitive binding data are combined with selectivity controls, and where biological claims are made, cellular localization or target-engagement evidence. This distinction is essential because complexes with similar in vitro stabilization profiles may diverge markedly in cells as a consequence of uptake, aggregation, ligand exchange, redox stability, protein binding, and subcellular distribution [14,17,30,31].

### 2.2. i-Motifs and Other Non-Canonical DNA Motifs

**Figure 3:**
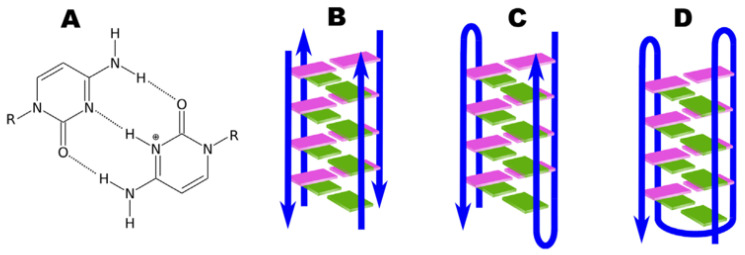
Structural organization of i-motif architectures. (**A**) Hemiprotonated cytosine–cytosine (C·C^+^) base pair. (**B**) Tetramolecular i-motif assembly. (**C**) Dimeric i-motif structure. (**D**) Intramolecular i-motif conformation. Reproduced from Reference [15] under a Creative Commons CC-BY license.

Compared with G4s, i-motifs (iMs, Figure 3) are a biologically important but less mature target class for noble-metal chemistry. Their stabilization depends on hemiprotonated C·C^+^ base pairs and is highly sensitive to pH, crowding, sequence context, chromatin accessibility, and protein readers. Recent genomic and cellular studies support context-dependent iM formation and regulation, but the direct evidence that noble-metal complexes selectively engage iMs in cells remains limited; therefore, iM examples are treated here as emerging rather than established [15,32,33].

Two mechanistic directions are particularly relevant, but they should not be conflated. Fluorescent Ag(I) biscarbene complexes demonstrate that silver-based scaffolds can discriminate selected non-canonical DNA structures through coordination and shape complementarity [34]. Ag^+^/H^+^-responsive systems show that metal ions can remodel cytosine-rich topologies [35]. However, the recent study by Seiffert and co-workers indicates that contiguous C-Ag(I)-C formation is generally not compatible with a canonical i-motif framework and may instead promote structural rearrangement [36]. Thus, Ag(I)-mediated cytosine pairing is best described as a topology-remodeling mechanism whose compatibility with iM biology must be established case by case.

Recent studies sharpen this conclusion and also caution against overgeneralization. The review by Sengupta and Sabouri summarizes the cellular i-motif field as increasingly compelling but still technically constrained by antibody specificity, temperature, pH, chromatin accessibility, and protein readers [33]. In parallel, Seiffert and co-workers directly asked whether the hemiprotonated C·C^+^ pairs of an i-motif/duplex junction can be replaced by C-Ag(I)-C base pairs and showed that compatibility is structure-dependent rather than automatic [36]. These results refine, rather than simply expand, the Ag(I)-i-motif picture: silver coordination can remodel cytosine-rich DNA, but evidence for biologically relevant i-motif targeting requires direct structural validation under the specific sequence, pH, and ionic conditions used.

Beyond i-motifs, the spectrum of non-canonical DNA targets is expanding. Quadruplex–duplex hybrid (QDH) structures create interfacial binding pockets absent in isolated G4s. Organic–platinum hybrids such as L^1^Pt(dien) and L^2^Pt(dien) exploit these interfaces through a combination of π-stacking at the G4–duplex junction and groove-oriented interactions, illustrating how selectivity can arise from composite structural features rather than single motifs [37].

Higher-order DNA architectures further broaden this landscape. Organometallic pillarplexes based on Au and Ag can bind and reorganize four-way Holliday junctions and related fork structures, demonstrating that metal-mediated recognition extends beyond grooves and tetrads to complex junction geometries [38].

More generally, motifs such as triplexes, mismatches, bulges, and junctions remain underexplored but are increasingly recognized as chemically addressable targets [39]. Taken together, these studies support a motif-resolved view of non-canonical DNA recognition in which G4s remain the dominant and best-validated target class, whereas i-motifs, QDHs, Holliday junctions, and related architectures should presently be regarded as emerging targets supported by a more limited body of evidence.

A further conceptual advance comes from locus-directed chemical biology rather than from metal coordination itself. Chemically modified dCas9 platforms carrying G4 or i-motif ligands now allow individual alternative DNA structures to be targeted in living cells, revealing that c-MYC G4 targeting can suppress promoter-specific transcripts, whereas targeting the opposite-strand i-motifs can have distinct transcriptional consequences [18]. Although this approach is not based on noble metals, it defines the level of locus resolution that future metal-based probes and conjugates should aim to reach when claiming structure-specific transcriptional control.

### 2.3. Structured RNA as an Expanding Frontier

Structured RNA represents an important but comparatively less consolidated frontier for noble-metal recognition. To keep the scope aligned with the review title, this section does not attempt a general survey of RNA structural biology. Instead, it focuses on RNA motifs for which metal binding, drug-induced RNA damage, ribonucleoprotein context, or fold-sensitive recognition is directly relevant to noble-metal chemistry [13,29,40,41,42,43,44,45,46,47,48,49,50,51,52,53,54,55].

**Figure 4:**
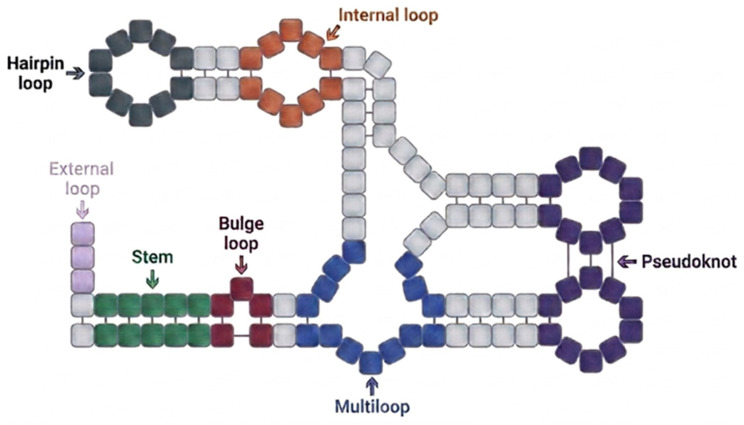
Representative non-canonical RNA structural motifs. Schematic representation of major RNA secondary and tertiary architectures, including hairpins, bulges, internal loops, multibranch loops, pseudoknots, and recurrent long-range interaction motifs. These elements contribute to the conformational and functional complexity of RNA and generate diverse recognition sites for metal-based ligands.

**Figure 5:**
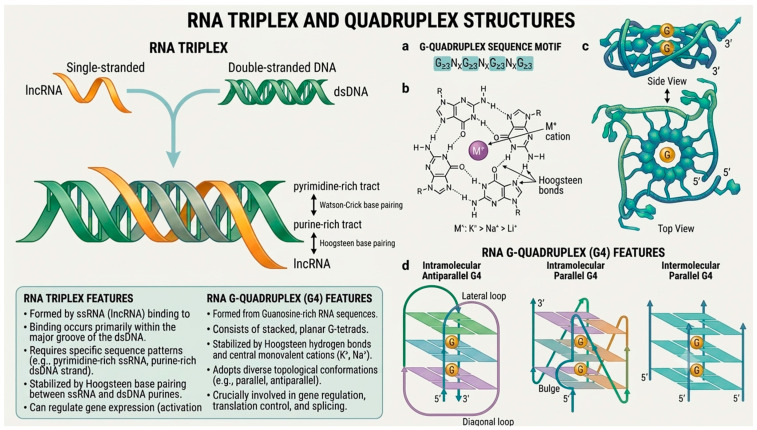
Representation of triplex and quadruplex RNA structures with their most relevant features.

RNA hairpins, internal loops, bulges, multibranch loops, pseudoknots (Figure 4), RNA G4s, and triplexes (Figure 5) create local asymmetry, accessible donor atoms, stacking surfaces, and electrostatic environments that can, in principle, support metal recognition. The key distinction, however, is evidential: these motifs are chemically plausible targets, but only a smaller subset has been examined with noble-metal complexes under conditions that allow direct binding modes to be assigned [13,29,43,44,45,46,47,48,49,50,51,52,53,54].

Rather than listing all RNA tertiary motifs in detail, the relevant point for this review is that recurrent RNA architectures can change donor accessibility and residence time for square-planar or soft metal centers. This is especially important for flexible internal loops and hairpins, where Pt(II) coordination to exposed guanine N7 sites can differ from the canonical duplex-DNA cross-linking paradigm [49,50].

Among higher-order RNA structures, RNA G4s and RNA triplexes are the most directly relevant to the present topic because they combine defined folding motifs with regulatory roles in translation, localization, and long non-coding RNA function [56,57,58,59]. At present, however, the noble-metal literature provides fewer direct structure-selective examples for RNA G4s and triplexes than for DNA G4s.

R-loops and Z-RNA are retained in the discussion only as RNA-containing or RNA-related alternative nucleic acid states. R-loops consist of RNA–DNA hybrids with a displaced DNA strand and are important in transcriptional regulation and genome stability, whereas Z-RNA is a left-handed RNA conformation linked to ADAR recognition and innate immune signaling [60,61,62,63,64]. These states should not be interpreted as ordinary structured-RNA targets for noble-metal complexes unless direct binding evidence is provided.

**Figure 6:**
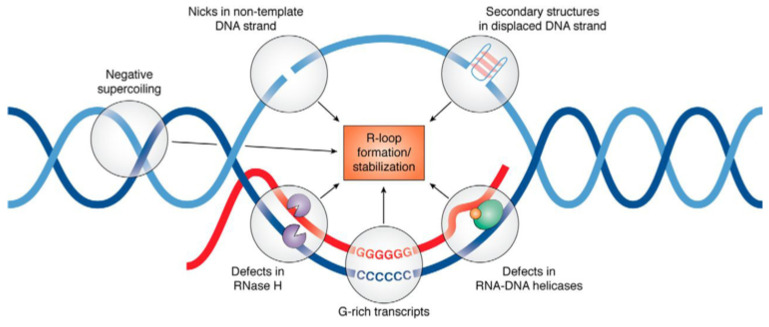
Structural organization of an R-loop. Reproduced from Reference [62] under a Creative Commons CC-BY license.

This distinction is important because, at present, R-loops (Figure 6) and Z-RNA mainly provide biological context and future target hypotheses rather than well-validated noble-metal binding platforms.

Furthermore, RNA can adopt alternative helical conformations such as Z-RNA, a left-handed structure analogous to Z-DNA, and recognized by ADAR family proteins, thereby linking RNA structural transitions to innate immune signaling and interferon responses [63,64]. Taken together, these features indicate that RNA should not be interpreted as a collection of isolated motifs, but rather as a dynamic ensemble of interconverting structures spanning local irregularities, tertiary folds, and hybrid nucleic acid states. This structural heterogeneity is directly relevant for metal-based recognition, as it generates a broad spectrum of coordination sites, stacking platforms, and electrostatic environments that differ substantially from those of canonical duplex nucleic acids.

Importantly, the same metal complex can exhibit markedly different behavior toward DNA and RNA targets, reflecting their distinct structural and dynamic properties. RNA, in particular, provides a richer landscape of non-canonical motifs—such as internal loops, bulges, pseudoknots, and higher-order folds—that modulate both accessibility and coordination modes. This is especially evident for square-planar d^8^ metal centers. While classical platinum(II) compounds have been historically associated with DNA binding, increasing evidence shows that Pt(II) complexes can efficiently target RNA, preferentially coordinating at flexible and solvent-exposed sites such as internal loops and hairpins, often involving N7 donor atoms of guanine residues. These features are enriched in non-canonical RNA architectures and promote platination patterns that differ from those observed in duplex DNA [13,49,50]. Comparative studies with Pd(II) complexes further indicate that variations in donor set, overall charge, and ligand framework can significantly redistribute binding affinity across DNA G-quadruplexes, RNA duplexes, RNA triplexes, and competing biomolecular partners such as albumin. In particular, square-planar Pd(II) systems bearing aromatic diimine ligands have been reported to preferentially stabilize G-quadruplex structures—primarily in DNA systems, but with increasing evidence in RNA contexts. Notably, relatively small variations in ligand design can shift the balance between nucleic acid binding and protein association under biologically relevant conditions, emphasizing the importance of speciation and competitive binding equilibria [51].

Ribosomal nucleic acids provide a particularly relevant RNA-centered context. Both rDNA and rRNA G-quadruplexes appear to adopt predominantly parallel topologies; however, RNA structures are generally more dynamic and less conformationally constrained than their DNA counterparts. This intrinsic flexibility is likely to influence both the residence time and selectivity of metal complexes, particularly within the crowded nucleolar environment. In this regard, beyond classical Au-based systems, ruthenium complexes have emerged as effective scaffolds for targeting structured RNA domains, combining shape-selective recognition with tunable coordination chemistry. For example, Ru-containing architectures have been shown to bind highly conserved ribosomal motifs such as the sarcin–ricin loop through a combination of non-covalent recognition, and when coupled to Pt centers, covalent anchoring. This highlights a synergistic strategy for stabilizing non-canonical RNA folds within ribonucleoprotein assemblies [29].

Beyond isolated nucleic acid targets, RNA recognition must also be considered within ribonucleoprotein assemblies and competitive biomolecular environments. Emerging proximity-labeling approaches suggest that metal complexes may perturb not only structured RNA elements, but also the local interaction networks in which these motifs are embedded [52].

In this context, the ability of certain metal ions to engage in non-canonical base pairing becomes particularly relevant. Ag(I), for example, is known to stabilize mismatched base pairs and unconventional pairing geometries through metal-mediated coordination, thereby reshaping local RNA structure and potentially modulating protein recognition interfaces. Such effects are especially significant in structurally heterogeneous RNA regions, where mismatch stabilization can alter folding equilibria and functional accessibility.

From a biological perspective, RNA targeting has the potential to broaden the functional scope of metal-based recognition. However, compared with promoter DNA G-quadruplexes, the mechanistic links between RNA binding, target engagement, and downstream phenotypic effects remain less extensively validated and require further investigation. Platinum-based drugs, for instance, have been shown to bind RNA at the transcriptome-wide level in cellular environments, suggesting that RNA may represent a major, yet underappreciated, pharmacological target. This view is supported by emerging methodologies such as G4-targeting biotin ligases and live-cell proximity labeling, which increasingly frame metal complexes as modulators of nucleic acid–protein networks rather than simple static binders. Moreover, the integration of structure-recognition elements (e.g., Ru-based scaffolds) with covalent binding units (e.g., Pt centers) points toward a new generation of metallodrugs designed to exploit the structural plasticity of non-canonical RNA [53,54].

Recent RNA-damage methodology further supports this cautious framing. AquIRE was developed to quantify drug-induced RNA damage and showed that clinically used agents, including oxaliplatin, can produce widespread and temporally dynamic RNA lesions while also enabling the detection of glycoRNAs [55]. This does not prove the selective binding of noble-metal complexes to a defined RNA fold, but it demonstrates that RNA can be a chemically and pharmacologically relevant target layer. For noble-metal studies, such approaches may help distinguish direct RNA modification or recognition from secondary transcriptomic stress responses.

## 3. Binding Modes and Physicochemical Determinants

**Scheme 3:**
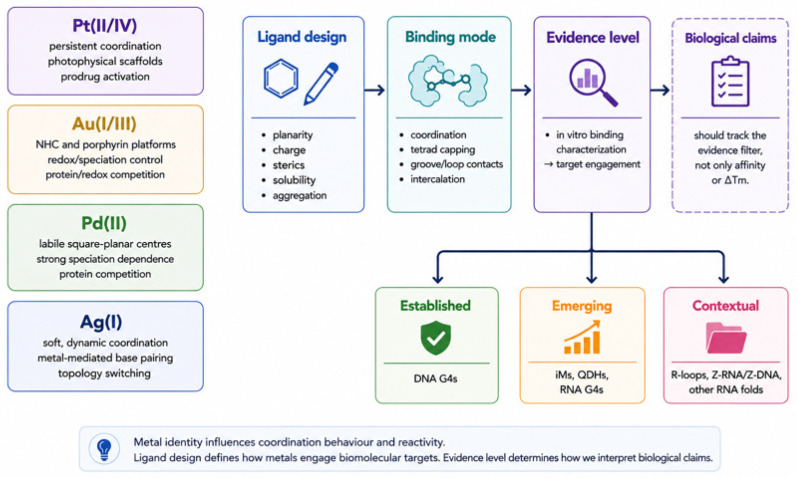
Overview of the relationships between metal identity, ligand design, binding mode, and evidence level in studies targeting non-canonical nucleic acid structures. The scheme highlights how metal-dependent properties and ligand features influence molecular recognition and the interpretation of biological claims across established, emerging, and contextual nucleic acid targets.

The following binding modes are presented as chemically distinct but experimentally interconnected categories. Scheme 3 summarizes the logic used in this review: metal identity constrains coordination kinetics and speciation, ligand design shapes the accessible binding mode, and the strength of the biological interpretation depends on the evidence filter applied to each system. Most detailed mechanistic information still derives from DNA G4s; other motifs are discussed where they illustrate emerging or contrasting behavior.

### 3.1. Coordination-Driven Recognition

Direct coordination remains one of the most chemically distinctive features of metal-based nucleic acid recognition. Pt(II) and Pd(II) complexes, owing to their square-planar geometry and accessible substitution chemistry, can coordinate nucleobase donor atoms such as guanine N7. In canonical duplex DNA, this underlies the classical cross-linking paradigm, but in non-canonical structures, the same chemistry is reshaped by donor accessibility, loop architecture, and local topology.

Reduction-activated Pt(IV)–salphen systems provide a clear illustration of this behavior. The prodrug is relatively inert, but after reduction, the released Pt(II)–salphen can stabilize G4 DNA through a combination of terminal π-stacking, electrostatic interactions, and hydrogen bonding, with the balance depending on protonation state [65].

**Figure 7:**
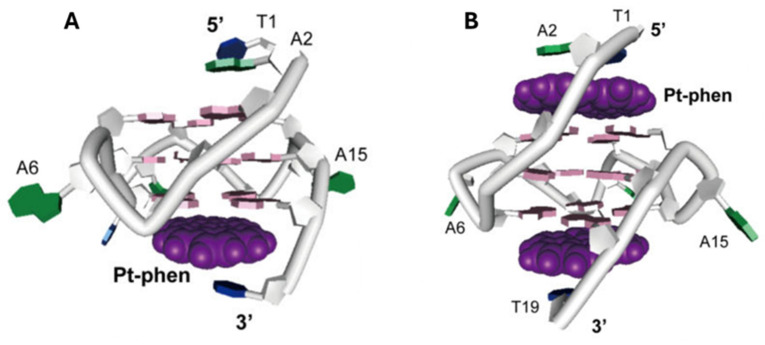
Stereo view of the representative structure of the (**A**) 1:1 and (**B**) 2:1 3′-end binding Pt-phen−MYC G4 complex (PDB Code: 9B6Z). Reproduced from Reference [24] under a Creative Commons CC-BY-NC license.

In G4 systems, coordination is rarely an isolated interaction channel. The Pt-phen complex bound to the MYC promoter G4 shows how coordination-compatible positioning and ligand-driven stacking operate together: the platinum chromophore sits over the tetrad center while flanking nucleotides reorganize to produce a tightly integrated adduct. Notably, binding proceeds sequentially, with the formation of a defined 1:1 complex at the 3′ end followed by a 2:1 adduct involving the 5′ end, highlighting the adaptive and end-specific nature of G4 recognition (Figure 7) [24]. A similarly non-unique binding landscape is observed for Au(III)-TPymT-α with telomeric G4, where multiple nearby poses are populated rather than a single rigid coordination geometry [28].

Softer metal centers such as Ag(I) and Au(I) typically display more labile and reversible interactions with nucleobase donors. This behavior makes them particularly suited for dynamic recognition and switchable systems. The terbium–platinum probe demonstrates how selective Pt–N7 coordination can be transduced into luminescent sensing, whereas Ag^+^-mediated i-motif remodeling shows that metal coordination can actively reshape nucleic acid architecture rather than simply bind a preformed structure [3,35].

Coordination-driven recognition becomes especially specific in hybrid architectures. In quadruplex–duplex hybrids such as the MYT1L system, L^1^Pt(dien) and L^2^Pt(dien) exploit composite binding pockets in which the aromatic ligand stacks at the G4–duplex interface while the Pt(dien) fragment engages the adjacent groove. This demonstrates that coordination chemistry and geometric complementarity can be co-optimized in ways not accessible for isolated G4 targets [37].

### 3.2. π-Stacking, Tetrad Capping, and External Association

For G4s, external π-stacking is often the dominant stabilizing interaction. Planar aromatic ligands associated with Pt, Au, or related metal centers can cap terminal G-tetrads, enhancing stability through dispersive, electrostatic, and desolvation-driven effects. This principle is shared across a wide range of systems, including Pt-phen, cyclometallated Pt(II) complexes, water-soluble Pt(II) luminophores, decorated organoplatinum c-MYC binders, AuTMX2, and Au(III) porphyrins [2,6,10,20,24,25].

However, tetrad capping alone does not account for true selectivity. Simple planar ligands may stabilize multiple quadruplexes with limited discrimination. Selectivity emerges when stacking is combined with additional structural elements, such as loop recognition, groove interactions, or multimeric interface binding. This is exemplified by dimeric salphen systems that preferentially target multimeric telomeric G4s, and by L^1^Pt(dien) and L^2^Pt(dien), which discriminate quadruplex–duplex hybrid architectures by exploiting interfacial pockets [8,37].

A particularly informative physicochemical case is provided by the Au(III) CNC pincer complex [Au(CNC)Cl]^+^ (4b). This system behaves as a partial intercalator rather than a classical full intercalator: spectroscopic and biophysical data support an intercalative orientation in duplex DNA, but steric encumbrance prevents deep insertion, leading to a non-canonical binding mode. This highlights that planarity alone does not dictate binding behavior; steric profile, charge distribution and ligand rigidity modulate the balance between tetrad capping, intercalation, and topology-selective association [66].

### 3.3. Groove, Loop and Side-Chain Interactions

Loops and grooves are often decisive elements for fine structural discrimination among non-canonical nucleic acid targets. In promoter G4s, variations in loop size and arrangement provide opportunities for selective recognition through ligand functionalization. In the c-MYC system, Pt(II) complexes bearing morpholine, amide, imide, or heteroaryl substituents adopt similar tetrad-stacking geometries but differ significantly in stabilization, optical response, and binding orientation depending on how the side chains engage loop and cap regions [2].

In telomeric systems, multinuclear Pt(II) complexes appear to rely more strongly on distributed groove interactions and extended electrostatic contacts across the quadruplex surface, illustrating an alternative strategy for achieving selectivity [7].

Some of these principles also appear to extend beyond canonical G4 targets to more complex architectures, although the supporting evidence is currently much more limited and often system-specific. In quadruplex–duplex hybrids, productive binding requires simultaneous engagement of an interfacial aromatic site and adjacent groove contacts. More generally, studies on Pd(II) complexes show that ancillary ligand design can shift the balance between nucleic acid binding, protein association, and cellular reactivity, indicating that peripheral interactions are key determinants of overall recognition behavior [51,67].

### 3.4. Multimodal Binding and Metal-Dependent Trends

Comparative studies consistently show that multimodal binding is the rule rather than the exception: Pt(II), Pd(II) and Au(I)/(III) complexes with related ligand frameworks may distribute their interactions across G4s, duplex DNA, selected RNA structures, and proteins, although the depth of mechanistic characterization remains much greater for G4-containing systems than for most alternative targets. Examples include organogold systems that combine moderate G4 stabilization with structural selectivity, Pd complexes where coordination chemistry is intertwined with serum-protein transport, and platinum probes whose optical response reflects both solvent shielding and productive nucleic acid association [9,16,25,51].

Broadly, metal identity plays a decisive role in shaping these interaction regimes. Pt(II) often provides a balance between persistent coordination and ligand-driven recognition. Pd(II), being more labile, is more sensitive to donor set and environmental competition. Au complexes, particularly in organometallic and NHC-based forms, tend to emphasize non-covalent recognition and multitarget behavior, while Ag(I) enables highly dynamic and reversible interactions, including topology switching in cytosine-rich structures.

In this context, physicochemical parameters such as speciation, redox stability, and resistance to biological nucleophiles become integral to understanding binding behavior. The stability of systems such as Au(III) CNC complexes toward glutathione, for example, is as mechanistically relevant as their intrinsic nucleic acid affinity, because only chemically persistent species can sustain productive interactions in cellular environments [66].

For this reason, mechanistic analysis should move beyond the simple question of whether a complex binds, and instead address how coordination, stacking, loop engagement, interfacial recognition, and speciation collectively define functional behavior. The most informative future studies will therefore integrate structural biology, orthogonal biophysical methods, imaging, interactome mapping, and cellular pharmacology, as already suggested by developments in live-cell G4-protein mapping and theranostic metal-based probes [30,52].

## 4. From Molecular Recognition to Functional Consequences

The transition from molecular recognition to biological effect is treated here as an evidence-graded problem rather than as a linear causal sequence. For selected G4-directed noble-metal complexes, the chain from fold recognition to telomerase inhibition, transcriptional modulation, mitochondrial dysfunction, or imaging can be assessed experimentally. For many other motifs, cellular phenotypes remain compatible with non-canonical nucleic acid engagement but are not by themselves proof of target occupancy (Table 3).

For clarity, the following section separates the experimentally demonstrated outcomes from mechanistic inferences. When cellular phenotypes are not supported by direct target-engagement data, they are described as compatible with, rather than proof of, non-canonical nucleic acid targeting.

Accordingly, biological outcomes are assigned higher confidence only when affinity/selectivity data are accompanied by localization, target engagement, pathway perturbation, or rescue-type evidence. Without such data, cytotoxicity, ROS formation, mitochondrial stress, or transcriptional changes are interpreted cautiously as downstream phenotypes that may involve several competing metal-dependent mechanisms [68,69].

**Table 3:** Representative noble-metal systems, target classes, evidence and limitations.

Metal/Scaffold	Primary Target or Model	Dominant Evidence	Biological Outcome	Key Limitation
**Pt(II) phenanthroline and cyclometallated Pt(II)**	Telomeric and promoter DNA G4s	ESI-MS, CD, melting, NMR or luminescence response [10,21,24]	G4 stabilization, cytotoxicity or imaging potential	Not all systems have cellular target-engagement proof.
**Trinuclear Pt(II) {[Pt(dien)]_3_(tib)}^6^^+^/{[Pt(dpa)]_3_(tib)}^6+^**	Human telomeric G4	Thermal stabilization, topology change, telomerase assay [7]	Telomerase inhibition	Focus mainly on telomeric models.
**Au(I) bis-NHC and Au(III) porphyrin/CNC systems**	DNA G4s and broader DNA structures	Biophysics, crystallography, modeling, cellular profiling [9,20,25,28,66]	Multimodal cytotoxicity, redox and mitochondrial effects	Nucleic acid targeting may compete with protein/redox mechanisms.
**Ag(I) biscarbene and Ag(I)-mediated cytosine systems**	Non-canonical DNA and i-motifs	Fluorescence, CD, structural and switching assays [34,35,36]	Dynamic topology remodeling	Physiological relevance depends on pH, sequence and Ag(I) availability.
**Pd(II) phenanthroline or palladate systems**	DNA/RNA models, G4s, i-motifs and proteins	Spectroscopy, competition, speciation and cellular assays [13,51,67,70]	Distributed nucleic acid/protein interactions	Lability and serum/protein binding complicate target assignment.
**Pt–salphen, PLIM Pt probes and radiolabeled Pt conjugates**	G4 DNA and cellular G4 imaging	Photophysics, PLIM, SPECT/CT and uptake studies [16,17,71]	Theranostic and imaging potential	Biodistribution or signal does not automatically prove intracellular G4 occupancy.

Bold entries in the first column denote the principal classes of noble-metal systems discussed in this review, whereas the accompanying columns summarize their primary targets, evidence base, biological outcomes, and current limitations.

### 4.1. Telomerase Inhibition, Telomere Dysfunction and Oncogenic G4 Control

The fan-shaped trinuclear Pt(II) complexes {[Pt(dien)]_3_(tib)}^6+^ and {[Pt(dpa)]_3_(tib)}^6+^ provide a clear example of how selective recognition translates into function. These systems preferentially bind human telomeric G4s over promoter G4s and duplex DNA, significantly increase telomeric G4 thermal stability, and inhibit telomerase with low-micromolar IC_50_ values, establishing a direct connection between structural selectivity and enzymatic inhibition [7].

A more advanced implementation of this strategy is found in the chiral Ru(II)–Pt(II) dinuclear complexes Δ-RuPt and Λ-RuPt. These compounds retain strong G4 stabilization under molecular crowding conditions, inhibit telomerase activity in cisplatin-resistant cells, and induce telomere dysfunction, apoptosis, and in vivo antitumor effects when delivered through biotin-functionalized DNA cages. This work is particularly significant because it bridges the gap between in vitro biophysical conditions and the crowded intracellular environment [72].

Temporal and chemical control over G4 targeting can also be engineered. In rotaxane-based systems, Pt(II)–salphen G4 binders are mechanically caged, showing reduced DNA affinity in the protected state and becoming highly cytotoxic only upon light- or esterase-triggered release, which is accompanied by nuclear localization and DNA engagement. Similarly, redox-activated Pt(IV)–Sal complexes act as prodrugs that release active Pt(II)–Sal species upon reduction, coupling conditional activation with quadruplex targeting [65,73].

Promoter G4s provide an additional layer of functional selectivity. The organoplatinum N^N^C series studied by Savva et al. displays a strong preference for the c-MYC G4 over telomeric and control sequences, combining end-stacking with side-chain interactions at loop regions. This demonstrates that oncogene regulation can arise from a refined recognition code involving both planar aromatic interactions and peripheral contacts [2,74].

### 4.2. Replication Stress, DNA Damage Signaling, and Apoptotic Commitment

A second functional axis emerges when non-canonical nucleic acid recognition intersects with DNA damage pathways. In this context, biological output is determined not only by binding, but by the ability of a complex to generate replication barriers, activate checkpoint responses, and drive apoptotic commitment.

The Au(III) CNC pincer complex [Au(CNC)Cl]^+^ (4b) is particularly illustrative. This system is redox-stable toward glutathione, displays significant cytotoxicity in breast cancer cells, and behaves as a partial DNA intercalator based on electrophoretic, spectroscopic, and hydrodynamic analyses. Computational studies further suggest its ability to recognize DNA three-way junctions and Z-DNA, indicating that its activity may arise from targeting structurally vulnerable DNA regions rather than a single canonical motif [66]. Notably, in this case, the mechanistic picture remains broader and less target-resolved than in the best-characterized G4-directed systems.

Cyclometallated Pt(II) complexes reported by McGhie et al. reinforce this principle. Their planar, luminescent scaffolds stabilize G4 structures, particularly H-Telo, and the most active compounds exhibit cytotoxicity exceeding that of cisplatin across multiple cell lines. These observations suggest that effective quadruplex recognition can translate into antiproliferative activity through the disruption of telomere maintenance and associated DNA processing pathways [10].

Other platinum systems highlight the importance of coordination environment and speciation in shaping biological output. In the TioxAla series, only selected complexes (Pt2 and Pt3) display both cytotoxicity and measurable interaction with different DNA conformations, indicating that ligand disposition and chloride retention critically influence activity [75]. In contrast, simpler systems such as [PtLCl_4_] or indole-derived Pt and Pd complexes exhibit DNA binding dominated by electrostatic or intercalative interactions toward CT-DNA, suggesting a less selective and more diffuse conversion of binding into biological effect [76,77].

Silver-based systems can converge on similar outcomes through different mechanisms. In Ag(I)–NHC complexes, selective cytotoxicity toward cancer cells is associated with ROS-mediated apoptosis, illustrating that the metal-induced perturbation of nucleic acid processing and redox balance can lead to common apoptotic endpoints even in the absence of highly specific G4 targeting [78].

### 4.3. Mitochondrial Dysfunction and Metabolic Reprogramming

Functional consequences are not confined to the nucleus. For several noble metal complexes, particularly gold and selected platinum systems, mitochondrial dysfunction represents a major downstream effect, expanding the mechanistic scope beyond nuclear DNA targeting.

**Figure 8:**
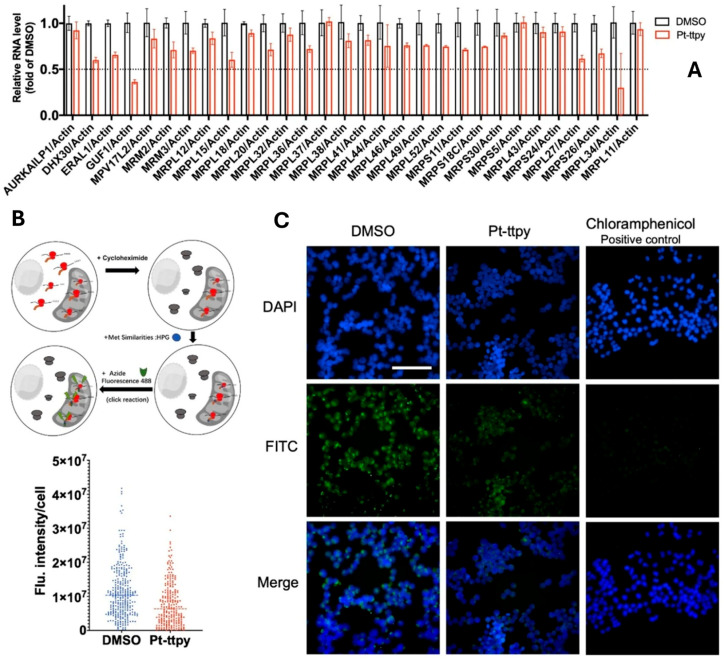
Impact of Pt-ttpy on nuclear and mitochondrial gene expression and mitochondrial translation. Pt-ttpy perturbs the transcriptional program of nuclear-encoded mitochondrial ribosomal genes enriched in G-quadruplex motifs and impairs mitochondrial protein synthesis in A2780 cells. (**A**) RT-qPCR analysis shows a broad downregulation of mitochondrial ribosomal gene expression upon Pt-ttpy treatment (mean ± SEM, *n* = 3). (**B**) Schematic illustration of studying flow of mt ribosome function by the Click-chemistry based IF assay. Left: down: Single-cell quantification showed Pt-ttpy significantly inhibited mitochondrial translation, DMSO group (>200), Pt-ttpy group (*n* > 200). Data represents three independent experiments. (**C**) (Representative fluorescence images illustrate reduced mitochondrial protein synthesis upon Pt-ttpy treatment, with chloramphenicol included as a positive control for the inhibition of mitochondrial translation (40× magnification). Reproduced from Reference [12] under a Creative Commons CC-BY license.

Pt-ttpy provides a clear example of this dual targeting behavior. This G4-binding platinum complex accumulates efficiently in mitochondria, induces mtDNA damage and copy-number reduction, suppresses mitochondrial transcription and translation, and impairs oxidative phosphorylation and oxygen consumption without significant ROS generation (Figure 8). Notably, it also downregulates nuclear-encoded mitochondrial ribosome genes enriched in promoter G4 motifs, establishing a link between mitochondrial and nuclear G4 targeting [12].

Gold complexes further broaden this perspective. Many Au(I)– and Au(III)–NHC systems exert biological activity primarily through mitochondrial pathways and the inhibition of thioredoxin reductase, with nucleic acid recognition contributing within a broader redox and organelle-centered mechanism [79,80].

Metabolomic studies reinforce the systems-level nature of these effects. Comparative analysis of different gold compounds, including auranofin and Au(NHC) derivatives, reveals distinct yet partially overlapping metabolic signatures, with significant perturbations in glycolysis, glutathione metabolism, and TCA cycle pathways. These findings demonstrate that closely related compounds can reprogram cancer metabolism through different network-level responses [81].

Palladium systems may contribute to this broader landscape through distributed interactions. Studies on palladate complexes such as 1-Ind and 2-All show preferential interactions with RNA helices, G4s, and i-motifs under varying conditions, suggesting that functional output may arise from simultaneous perturbation of multiple nucleic acid pools rather than a single dominant lesion [70].

### 4.4. Functional Consequences Are Shaped by Speciation, Protein Binding and Pharmacological Context

A central conclusion emerging from these studies is that biological outcome is strongly filtered by pharmacological context. Even structurally competent binders may lose, redistribute, or redefine their activity in biological environments because speciation, ligand exchange, protein binding, uptake, and subcellular trafficking determine which species actually reaches the intended target [69].

This is particularly evident for platinum drugs in the blood. Clinically used Pt agents differ substantially in plasma protein binding, reversibility of adduct formation, half-life and renal clearance, all of which influence transport, deactivation, and toxicity. These parameters directly determine how much of an active species can reach nuclear or mitochondrial targets [82]. Importantly, this competitive landscape extends well beyond plasma proteins. Inside cells, thiols, other biological nucleophiles, membrane partitioning, and organellar sequestration can all divert, inactivate, or redistribute metal complexes, thereby eroding or redefining the apparent selectivity inferred from simplified in vitro systems.

Even in simpler DNA-binding systems, this principle is apparent. Indole-3-acetic-acid-derived metal complexes display measurable DNA affinity and anticancer activity, yet their interaction is largely dominated by intercalative binding to CT-DNA rather than the selective recognition of defined non-canonical structures. Such systems remain informative but highlight the need to distinguish between general DNA binders and truly structure-selective agents [77].

Taken together, these studies indicate that the progression from molecular recognition to biological effect is not linear but modulated by activation pathways, structural selectivity, protein interactions, intracellular trafficking, and metabolic adaptation. The most promising next-generation systems are therefore likely to combine precise structural recognition with controlled activation and a clearly defined systems-level biological response.

## 5. Systems-Level Effects: Proteomics, Metabolomics and Metallomics

A review focused only on direct metal-nucleic acid binding would miss an essential part of metallodrug biology, but omics data also require careful interpretation (Table 4). Proteomics, metabolomics, and metallomics are therefore used here as mechanistic filters: they can support, refine, or contradict a structure-based hypothesis, but they do not replace direct evidence that a metal complex engages a specific nucleic acid fold in cells [68,83].

### 5.1. Proteomics and the Reframing of Mechanism

Proteomic analyses have been particularly influential in the case of gold compounds, where they helped dismantle the simplistic expectation that all cytotoxic noble metal agents should behave like platinum drugs. The most systematic study in this area examined a representative panel of gold compounds in A2780 ovarian cancer cells, including auranofin, Auoxo6, Au_2_phen, AuL12, Aubipyc, Au(NHC)Cl, and [Au(NHC)_2_]PF_6_, by combining 2-DE, MALDI-TOF identification, and bioinformatic interpretation. Across this series, recurrent perturbation of redox-control systems and proteasome-related pathways emerged as dominant signatures, while Aubipyc and [Au(NHC)_2_]PF_6_ produced broader remodeling that also involved glycolytic enzymes and carbon metabolism [83]. This body of work is highly relevant here because it shows that for noble metal compounds, the primary executory phenotype may be protein-centered even when nucleic acid recognition remains chemically plausible.

The conceptual value of proteomics is that it reframes the mechanism from a single binding event to a pathway response. In the gold series above-discussed, the strongest signals did not simply indicate generic stress, but converged on tractable nodes such as thioredoxin-dependent redox balance, proteasome function, and metabolic rewiring, thereby helping to distinguish compound-specific from class-wide effects [83]. This systems view becomes especially important for putative G4-targeting agents, because even a genuine quadruplex interaction may operate only as the initiating lesion within a wider adaptive network involving nucleolar stress, mitochondrial dysfunction, cytoskeletal remodeling, and proteostatic compensation.

For platinum compounds, proteomics adds a different but equally important layer of information. Mass-spectrometric studies of protein binding in blood have shown that cisplatin and its analogues rapidly associate with serum proteins such as albumin, transferrin, globulins, and hemoglobin, with direct consequences for transport, deactivation, renal clearance, and systemic toxicity [82]. Thus, even before a Pt complex reaches a nuclear or mitochondrial G4, its biological identity may already have been reshaped by the proteome. In other words, protein binding is not merely background pharmacology, but part of the mechanism that controls exposure of any non-canonical nucleic acid target.

Taken together, these proteomic observations argue that structure-based claims should be interpreted together with pathway-level evidence. A noble metal complex may bind a G4 *in vitro* and still behave mainly as a redox or proteostasis modulator in cells; conversely, a modestly selective binder may become biologically powerful if it perturbs a protein network that is functionally coupled to the targeted fold. Proteomics is therefore indispensable for ranking mechanisms rather than merely listing possible targets.

### 5.2. Metabolomics and Bioenergetic Rewiring

Metabolomics is particularly well-suited to detect the downstream consequences of organelle stress, redox imbalance, and translational defects. It therefore provides a natural complement to structural studies on non-canonical nucleic acid targeting. If a metal complex perturbs a mitochondrial G4, for example, the resulting phenotype should not stop at a change in folding equilibrium; it should propagate into TCA cycle flux, amino acid usage, redox poise, and biosynthetic allocation.

Recent modeling and experimental work on cytotoxic gold compounds in ovarian cancer cells illustrates this principle particularly well. By integrating NMR metabolomics with a context-specific genome-scale metabolic model of A2780 cells, Vieri and co-workers showed that auranofin, aurothiomalate, Au(NHC) and Au(NHC)_2_ all induce major but non-equivalent metabolic responses. Auranofin was associated with a marked increase in glutathione and only a limited rise in lactate; the two gold N-heterocyclic carbenes strongly increased glycolytic output and lactate production; aurothiomalate preferentially perturbed TCA-related metabolism without the same glycolytic signature. The model reproduced around 70% of the experimentally observed metabolite changes and highlighted pathway-level responses involving glutathione handling, fatty-acid beta-oxidation, transport processes, and the GDP-L-fucose node, supporting the idea that gold-based cytotoxicity often relies on metabolic disruption rather than direct DNA damage alone [81].

For the present review, the implication is straightforward. Even when noble metal complexes are designed to recognize a specific non-canonical nucleic acid structure, the biologically decisive outcome may be a systems-level reallocation of metabolic flux rather than a persistent structural lesion per se. Metabolomics therefore serves as the functional readout of target engagement, while computational modeling helps identify which parts of the metabolic network are causally reprogrammed and which are merely downstream consequences [31,81,84].

### 5.3. Metallomics, Metal Trafficking and Subcellular Distribution

Metallomics provides the missing physical dimension of metallodrug biology: where the metal goes, in what chemical form it resides, and which compartments accumulate biologically meaningful amounts. Without this information, structure-based mechanism proposals remain incomplete. A complex designed to target a promoter G4 will fail mechanistically if it is rapidly trapped by serum proteins, exported, reduced to a different species, or partitioned into lysosomes.

A clear example of the mechanistic importance of metal trafficking comes from fludarabine-derived palladium and platinum complexes bearing trans-[Br(PPh_3_)_2_]M fragments. In that series, the platinum compounds **3b** and **4b** were markedly more cytotoxic and far more selective for tumor over non-malignant cells than the palladium analogues **3a** and **4a**, although the palladium complexes bound calf thymus DNA more readily. ICP-MS showed that the platinum species reached intracellular levels roughly one order of magnitude higher than the palladium congeners, whereas the palladium compounds displayed relatively greater DNA metalation once inside the cell. These results are mechanistically instructive: biological efficacy did not correlate with intrinsic DNA reactivity alone, but with cellular uptake, persistence, and the fraction of metal that actually reached productive intracellular compartments [85,86].

A complementary perspective is offered by radiometal-based imaging of quadruplex binders. Lo and co-workers functionalized a Pt(II)–salphen G4 binder with DOTA and radiolabeled it with ^111^In, obtaining a probe that retained the in vitro G4-binding profile of the parent Pt–salphen scaffold. SPECT/CT imaging in a melanoma mouse model showed rapid renal clearance together with measurable tumor accumulation and persistence of the radiolabeled complex **7**. Yet the same study is notable for its caution: poor cellular uptake indicated that tumor retention could not be interpreted as direct evidence of intracellular G4 engagement. This is an excellent example of how metallomics, imaging and pharmacokinetics sharpen mechanistic interpretation by separating biodistribution from target occupancy [16].

### 5.4. G4-Protein Interactomes and Functional Architectures

Perhaps the clearest conceptual justification for the term functional architecture lies in the G4-protein interactome literature. G4s are not isolated motifs but hubs that recruit, exclude, or exchange proteins involved in chromatin regulation, transcription, replication, RNA processing, and repair. Live-cell profiling of DNA G4-interacting proteins established this network view, while recent G4-ligand-based PROTACs show that endogenous chromatin G4s can be exploited to degrade proximal G4-binding proteins such as FUS, SMARCA4, and ATRX [19,87].

The newest G4L-PROTAC work further clarifies what a functional architecture can mean experimentally. In that study, bifunctional molecules based on a G4 ligand recruited E3 ligases to endogenous chromatin G4 sites and promoted the degradation of associated proteins, including FUS, SMARCA4 and ATRX, with proteasome dependence and chromatin-context selectivity [19]. Although these compounds are not noble-metal complexes, the study provides a rigorous experimental benchmark: future metal-based G4 probes that claim network-level action should ideally connect fold binding, chromatin localization, proximal protein perturbation, and selective downstream response in a similarly integrated manner.

This idea is reinforced by live-cell approaches identifying G4-interacting proteins linked not only to genome maintenance, but also to RNA processing and splicing. In this view, noble metal recognition of a G4 becomes biologically significant when it perturbs the local protein network organized around that fold.

This network view also resolves an apparent contradiction in the field. Why do some excellent G4 binders show modest biological impact, whereas others with similar in vitro stabilization values produce major cellular phenotypes? One answer is that functional activity depends on whether the ligand perturbs the right architecture—the right combination of nucleic acid fold, compartment, protein readership, and kinetic persistence. Omics approaches help identify these architectures, and noble metal chemistry offers tools to manipulate them with unusual precision.

## 6. Therapeutic Implications and Design Challenges

The translational promise of noble metal targeting of non-canonical nucleic acids is now more concrete because several recent Pt-based systems have begun to connect molecular recognition with imaging, photoactivation, and biodistribution. This section, therefore, emphasizes practical design constraints for metal complexes and metal-enabled probes rather than presenting a broad therapeutic survey. At the same time, these studies make clear that translational potential is not determined by G4 affinity alone, but by the broader convergence of fold selectivity, intracellular delivery, aqueous stability, photophysical behavior, dark-versus-light cytotoxicity, and retention of function after diagnostic or solubilizing modifications [6,10,16,17,30,71].

### 6.1. Selectivity Beyond Affinity

Selectivity remains the central medicinal challenge. The recent Pt–salphen study of Bartlett et al. shows this very clearly: across a 21-member library, good G4 affinity and selectivity over duplex DNA were relatively common, yet the most promising behavior emerged only for compounds that combined recognition with suitable uptake and photochemical competence. In particular, L2_4OMe and L3_4OMe stood out as practical leads because they retained good G4 selectivity, entered cells efficiently, showed some nucleolar localization, and were essentially non-cytotoxic in the dark while becoming strongly phototoxic upon irradiation. This moves the design discussion away from static binding constants toward functional selectivity under biologically relevant conditions [71].

A related lesson emerges from the water-soluble phosphorescent Pt(II) series of Kroos et al. Their sixteen N^N^C complexes demonstrate that improving water solubility and probe-like behavior is not a trivial add-on but a core design problem. The authors found a complex and sometimes counterintuitive relationship between substituent pattern, aggregation tendency, luminescence turn-on, and G4 responsiveness. Thus, a useful theranostic candidate must balance planarity and cationic character with steric control and sufficient polarity, while avoiding self-association that can blur both binding and imaging readouts. In practical terms, the solubilization strategy is part of pharmacology, not merely formulation [6].

This point is reinforced by the 2026 Pt-based phosphorescent lifetime probes developed for PLIM visualization of cellular G4s. Their value lies not simply in brighter emission, but in lifetime-based discrimination of quadruplex environments and in the reported absence of nuclear/nucleolar self-aggregation, which directly addresses artifacts that can undermine the intracellular interpretation of G4 probes [17].

### 6.2. Resistance, Adaptation and Target Plasticity

Resistance and adaptation should likewise be viewed through a structural lens. The cyclometallated Pt(II) CMC series of McGhie et al. illustrates that strong quadruplex stabilization, up to 19 °C in melting experiments, does not map perfectly onto a single biological endpoint. These complexes stabilized H-Telo more strongly than c-MYC, and the more active derivatives, especially complexes **6**, **7**, and **9**, outperformed cisplatin across multiple cancer cell lines while maintaining moderate selectivity. This suggests that the therapeutically relevant design space is multidimensional: target choice, quadruplex topology, timing of ligand addition relative to fold formation, and whole-cell uptake all contribute to outcome [10].

Target plasticity is also likely to be important. Because different promoter and telomeric G4s respond differently to the same scaffold, cells may buffer drug action by shifting the balance among accessible folds or by changing protein occupancy at those sites. This possibility makes combination strategies especially attractive. Photoactivatable systems could be paired with replication-stress or repair inhibitors, whereas non-photoactive G4 stabilizers might be combined with agents that limit adaptive folding or helicase-mediated rescue. The central point is that non-canonical nucleic acid targeting is best understood as dynamic intervention in a structural network rather than as occupation of a single immutable receptor.

### 6.3. The Shift Beyond the DNA-Centric Paradigm

**Figure 9:**
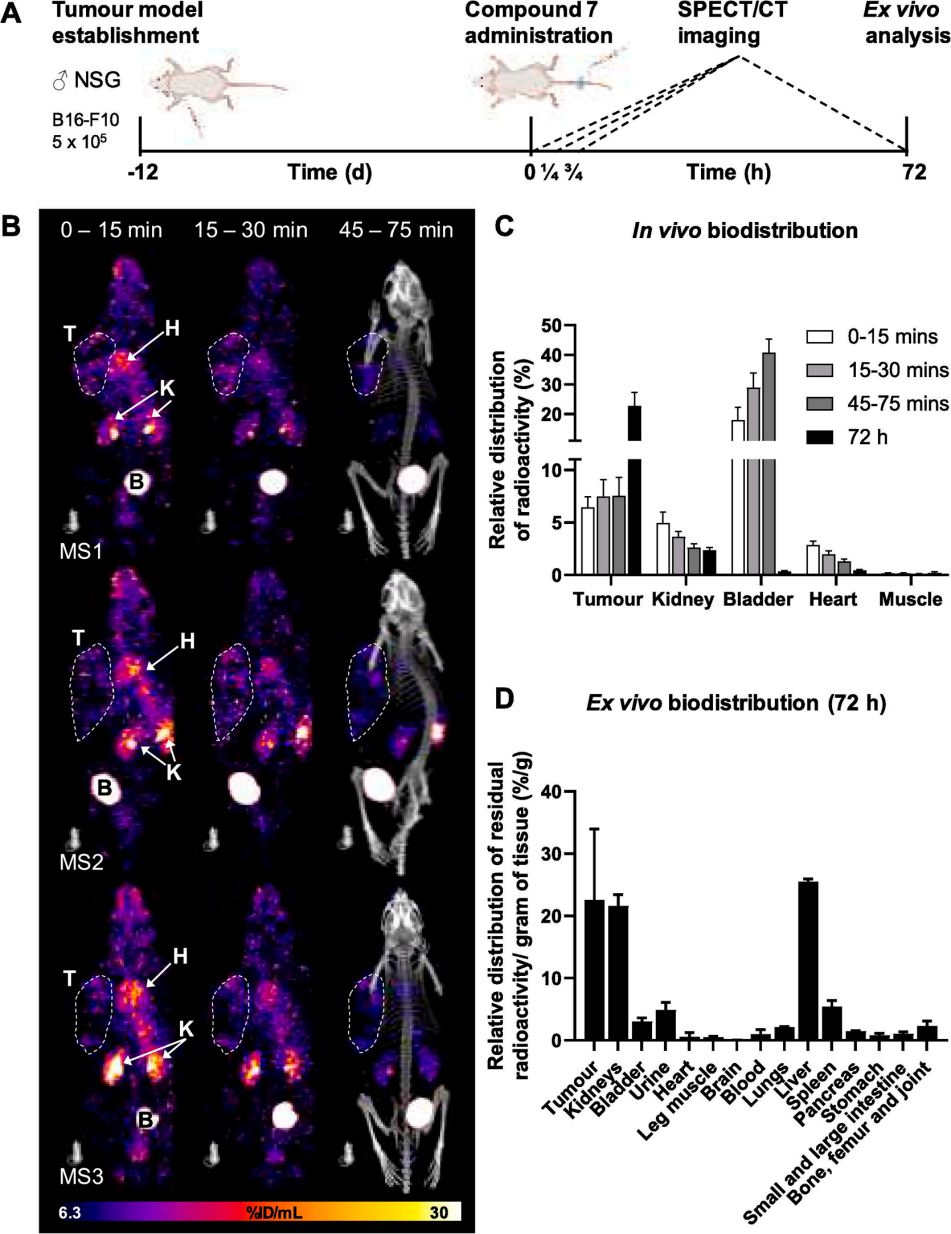
*In vivo* SPECT/CT imaging and biodistribution of ^111^In radiolabeled Pt(II)–salphen complex. (**A**) Schematic overview of the experimental design for *in vivo* imaging. (**B**) Maximum intensity projection (MIP) images obtained from SPECT alone and fused SPECT/CT at the indicated time points following administration of the compound (time ranges correspond to the duration of each full SPECT acquisition). Tumor regions (T) are identified on CT images and outlined with white dashed lines. Additional regions of interest are indicated by arrows: kidneys (K), heart (H), and bladder (B). (**C**) Quantitative analysis showing the percentage distribution of radioactivity across selected regions of interest relative to the whole body. (**D**) *Ex vivo* biodistribution of residual radioactivity expressed per gram of tissue, measured 72 h after administration (mean ± SD, *n* = 3). For *in vivo* imaging, bladder and urine signals were combined, whereas they were analyzed separately in *ex vivo* measurements. Reproduced from Reference [16] under a Creative Commons CC-BY license.

Another major lesson from these recent papers is that the classical divide between therapy and diagnosis is becoming less useful. Palma et al. framed this transition conceptually in their theranostics review, emphasizing that fluorescent metal complexes can already serve as G4-sensitive probes, and that the next step is to integrate these properties with clinically tractable imaging radionuclides. Lo et al. further illustrate the modular potential of this strategy: a Pt–salphen G4 binder can be equipped with a radiometal-compatible appendage while retaining a recognizably related *in vitro* binding profile. The broader lesson is not simply that imaging can be added, but that recognition, reporting, and delivery must be co-designed rather than treated as separable layers (Figure 9) [16,30].

At the same time, Lo et al. also exposed the current limits of translation. Their ^111^In conjugate accumulated in tumor tissue in vivo and enabled SPECT/CT biodistribution studies, but the corresponding non-radioactive probe showed poor cellular uptake, indicating that tumor retention was not sufficient evidence of intracellular G4 engagement. This is an important design warning for the field: *in vivo* localization, optical signal, and even favorable pharmacokinetics cannot by themselves be taken as proof of target occupancy. True theranostic progression will require concurrent optimization of cell permeability, subcellular delivery, and retained fold selectivity after radiolabel installation [16].

The broader implication is that functional architectures should now be designed modularly: the recognition scaffold, reporting function, activation mode, and delivery properties must be co-optimized from the outset.

The recently reported Pt-based phosphorescent lifetime probes by Bellamkonda and co-workers strengthen this translational direction because one probe showed lifetime changes that discriminated quadruplex DNA from duplex DNA or solution and enabled PLIM visualization of G4s in live and fixed cells without detectable nuclear or nucleolar self-aggregation [17]. This directly addresses a reviewer-relevant limitation of earlier emissive probes: a useful imaging readout must report fold-sensitive behavior while minimizing aggregation artifacts in the compartment where G4s are inferred to reside.

### 6.4. Clinical Translation and Future Design Principles

The path to clinical translation remains demanding, but the operative design principles are becoming clearer. Scaffolds must remain soluble, emissive and structurally selective under aqueous and intracellular conditions; dark and triggered toxicity must be intentionally separated when photoactivation is used; and imaging modules should be introduced early enough to verify whether pharmacokinetic optimization is compatible with meaningful cellular and subcellular target access [16,71].

Accordingly, future design should emphasize a small number of experimentally testable principles: topology-sensitive recognition instead of generic aromaticity; explicit control of solubility and aggregation; verification of cellular and subcellular uptake; orthogonal readouts linking imaging to target engagement; and comparative biological evaluation against specific folds such as c-MYC, H-Telo, c-kit, and bcl2-derived quadruplexes rather than against a generic G4 label. In this framework, selected Pt–salphen photosensitizers (Figure 9), water-soluble phosphorescent Pt(II) probes, radiolabel-compatible Pt–salphen conjugates, and cyclometallated cytotoxic Pt(II) complexes can be viewed as complementary prototypes for the next generation of noble-metal G4 theranostics [6,10,16,71].

## 7. Conclusions and Future Directions

Noble-metal coordination to non-canonical DNA and RNA structures is best viewed as a G4-centred field with expanding, but unevenly validated, frontiers. DNA G4s remain the benchmark because several Pt, Au, Pd, and Ag systems have been characterized by orthogonal structural and cellular methods. In contrast, i-motifs, RNA structures, R-loops, Z-DNA/Z-RNA, triplexes, quadruplex–duplex hybrids, and junctional architectures are promising but usually supported by fewer direct noble-metal target-engagement data. This revised framing narrows the apparent scope mismatch while retaining the broader conceptual relevance of emerging non-canonical architectures.

In the revised framework, the field is therefore explicitly G4-centred but not G4-exclusive. G4s remain the benchmark for mechanistic confidence, whereas i-motifs, QDHs, junctions, R-loops, Z-DNA/Z-RNA, and RNA structures define the frontier where stronger target-engagement standards are needed before broad biological claims are made.

A first major future direction concerns target definition. Many current studies still rely on a limited panel of model oligonucleotides, whereas the biologically relevant targets are dynamic, topologically heterogeneous, and strongly influenced by chromatin environment, molecular crowding, protein readers, and subcellular localization. Medium-term progress will require a shift from isolated structural motifs to functional architectures, that is, nucleic acid folds considered together with their protein context, compartmentalization, and temporal dynamics. Only through this type of convergence will it become possible to discriminate true on-target recognition from downstream stress responses or from generic metal-induced damage.

A second critical direction is molecular design. The most promising scaffolds emerging from the literature combine several features: extended yet not excessively bulky aromatic surfaces for end-stacking, controlled charge distribution, sufficient kinetic and redox stability in biological media, tunable ligand exchange properties, and when needed, appended groups that modulate uptake, organelle distribution or local secondary recognition. For platinum, gold, palladium, and silver systems alike, future design should move beyond empirical decoration and embrace mechanism-guided optimization. In practical terms, this will include designing families of congeners in which planarity, lipophilicity, steric demand, counterion effects, photophysical behavior and activation pathways can be varied independently. Particular attention should be paid to prodrug approaches, redox-activated systems, photoactivatable constructs, and radiolabeled derivatives because these strategies offer realistic routes to improve selectivity while preserving the unique advantages of metal coordination.

A third direction concerns biological translation. One of the strongest lessons from the reviewed literature is that anticancer activity rarely correlates in a simple way with G4 stabilization alone. Cellular uptake, serum stability, off-target protein binding, mitochondrial effects, redox imbalance, proteostasis perturbation, and metabolic rewiring all contribute to phenotype. Accordingly, future preclinical development should adopt a more rigorous progression scheme: biophysical characterization, validation in increasingly complex cellular models, systems-level profiling, confirmation of target engagement in cells, and only then in vivo assessment. Advanced tumor models, including organoids, co-culture systems, and resistant cell lines, will be particularly important to determine whether these compounds exploit vulnerabilities that remain inaccessible to conventional platinum drugs. In the medium-term, the most plausible practical applications are likely to arise in niches where standard therapies fail, such as resistant tumors, transcription-addicted cancers, telomere-maintenance-dependent settings, or tumors that may benefit from image-guided or externally activated interventions.

Finally, the theranostic dimension deserves special emphasis. Luminescent Pt(II) systems, photoresponsive salphen derivatives, and radiolabeled G4 binders already indicate that the same coordination scaffold can, in principle, support target recognition, mechanistic visualization, and therapy. This convergence is still at an early stage, but it may become one of the most productive trajectories of the field. If future compounds can achieve adequate tumor delivery, intracellular access, validated target engagement, and acceptable pharmacology, they may support practical applications such as companion imaging for patient stratification, real-time monitoring of biodistribution, or combined diagnostic-therapeutic platforms. Thus, the medium-term outlook is not simply the discovery of stronger binders, but the emergence of integrated metal-based systems able to connect molecular recognition with actionable biological and clinical information.

A final conceptual advance will be to distinguish more explicitly among metal-centered effects, ligand-driven recognition, and emergent properties of the whole construct. In many systems, biological activity does not arise from the metal ion, the ligand scaffold, or the nucleic acid target considered in isolation, but from their coupled behavior within a specific chemical and cellular context.

Reaching this goal will require close collaboration among coordination chemists, structural biologists, omics specialists, pharmacologists, and clinicians, but the conceptual foundations are now sufficiently mature to justify that effort.

Practically, future manuscripts in this area should report not only ΔTm or affinity values, but also speciation, aggregation behavior, matched-fold selectivity, cellular uptake, compartmental distribution, and at least one target-engagement or proximity readout whenever biological claims are made. Applying this evidence ladder will help distinguish genuine structure-selective noble-metal systems from general nucleic-acid binders or broadly cytotoxic metal compounds.

## Figures and Tables

**Table 1:** Evidence maturity across non-canonical nucleic acid targets discussed in this review.

Target Class	Direct Noble-Metal Binding Evidence	Structural/Biophysical Validation	Cellular Target Engagement	Biological Outcome	Current Maturity	Main Open Question
**DNA** **G-quadruplexes**	Extensive for Pt, Au, Pd, and Ag platforms [13,14,15]	Thermal melting, CD, ESI-MS, fluorescence, NMR/X-ray for selected systems, modeling	Demonstrated for selected compounds, especially telomeric, promoter and mitochondrial G4 systems	Telomerase inhibition, promoter regulation, mitochondrial dysfunction, cytotoxicity, imaging or theranostic prototypes	Established	Need more direct intracellular target engagement and fold-specific causal validation
**i-motifs**	Limited but growing, especially Ag-based or luminescent probes	pH-dependent folding assays, spectroscopy, selected coordination studies Recent cellular and Ag(I)-metalation studies emphasize both biological relevance and structural constraints	Scarce	Mostly inferred or model-system based	Emerging	Need physiological pH validation and cellular engagement studies
**Quadruplex–duplex hybrids**	Available for selected organic-Pt hybrid systems	Computational and biophysical studies on defined hybrid architectures	Limited	Potential selectivity for composite interfaces	Emerging	Need more structural and cellular validation
**Holliday junctions/forks**	Shown for selected organometallic pillarplexes	Structural and biophysical evidence for junction/fork recognition	Limited	Biological implications remain underdeveloped	Emerging	Need cell-based target engagement and functional assays
**RNA G-quadruplexes**	Supported mainly by analogies and selected RNA-focused studies	CD, spectroscopy, computational and comparative binding studies	Developing	Translation, localization and stress-response hypotheses	Emerging	Need direct noble-metal binding and transcript-specific validation
**Structured RNA motifs**	Direct Pt-RNA and Ru/Pt-RNA studies exist, but noble-metal examples remain less systematic	Mapping, cross-linking, spectroscopy and model RNA studies	Available in selected cases but not broadly fold-resolved	Potential modulation of RNA-protein assemblies Drug-induced RNA damage can be mapped, but structure-selective noble-metal engagement remains to be proven	Emerging	Need motif-specific selectivity and intracellular validation
**R-loops**	Currently mostly indirect for noble-metal targeting	Strong biological literature on R-loop structure/function, limited metal-binding evidence	Not established for noble-metal complexes	Genome stability relevance is high but metal-target link is speculative	Indirect/conceptual	Need direct binding and cellular perturbation evidence
**Z-DNA/Z-RNA**	Limited direct evidence for noble-metal recognition	General structural and biological literature exists	Not established for the systems discussed here	Potential relevance to immune signaling and alternative nucleic acid topology	Indirect/conceptual	Need direct noble-metal binding and functional validation

Bold text in the first column highlights the major categories of non-canonical nucleic acid targets discussed in this review.

**Table 2:** Operational evidence standards used to separate direct recognition from inferred or network-level effects.

Evidence Layer	Operational Criterion	Interpretation in This Review
**Direct structural recognition**	Binding to a defined fold shown by at least two orthogonal assays, e.g., CD/FRET/Tm plus MS, NMR, crystallography, footprinting or validated modeling.	Supported for several DNA G4 systems; less common for iMs, RNA and junctions.
**Selectivity**	Comparison against duplex DNA and at least one alternative non-canonical fold under matched conditions.	Often reported for G4s, but incomplete for many RNA and Ag(I)-iM examples.
**Cellular access**	Evidence that the active or emissive species reaches the relevant compartment without aggregation or dominant sequestration.	Critical for Pt PLIM probes and radiolabeled Pt–salphen systems [16,17].
**Target engagement**	In-cell signal, locus targeting, proximity mapping, pull-down, perturbation/rescue or degradation of proximal readers.	Emerging benchmark from ATENA and G4L-PROTAC studies [18,19].
**Functional architecture**	Coherent link among fold binding, protein/network context, localization/speciation and phenotype.	A high-confidence claim only when multiple evidence layers converge.

Entries shown in bold in the first column represent the evidence categories adopted in this review to distinguish direct structural recognition from progressively higher levels of biological validation and functional interpretation.

**Table 4:** Systems-level evidence layers and their interpretative limits.

Evidence Layer	What It Adds	Main Caveat
**Proteomics/interactomics**	Identifies proteins and pathways perturbed by a compound; can reveal G4-associated protein networks.	Does not prove that the initiating lesion is a specific nucleic acid fold unless proximity, competition or rescue data are included.
**Metabolomics**	Reports mitochondrial, redox and bioenergetic consequences of treatment.	Often downstream of multiple primary events; useful for phenotype, not sufficient for target assignment.
**Metallomics/imaging**	Measures uptake, biodistribution, metal accumulation and compartmentalization.	Accumulation in a tissue or organelle is not equivalent to target occupancy [16].
**Locus-specific targeting**	Tests a defined G4 or iM in its genomic context, as in dCas9-ligand approaches.	Currently mostly outside noble-metal chemistry but defines a benchmark for future metal conjugates [18].
**G4L-PROTAC strategies**	Perturb proteins associated with endogenous chromatin G4s.	Not metal-based, but provides an operational model for functional architecture claims [19].

Entries shown in bold in the first column represent the major systems-level evidence categories used to evaluate how molecular recognition events translate into cellular and biological outcomes.

## Data Availability

No new data were created or analyzed in this study. Data sharing is not applicable to this article.
